# Supplementary material for: Local organization of spatial and shape information in the primate prefrontal cortex
Source: Cereb Cortex. 2024 Sep 25;34(9):bhae384. doi: 10.1093/cercor/bhae384 (PMC11422719; doi:10.1093/cercor/bhae384)
Supplement: LocalPFCOrganizationSupplementary_2024_09_03_bhae384 [file localpfcorganizationsupplementary_2024_09_03_bhae384.pdf]

Supplementary Information for

Local organization of spatial and shape information in the primate prefrontal cortex

Yunyi Sun[1*], Wenhao Dang[2*], Rye G. Jaffe[2], Christos Constantinidis[2,3,4]

1. Department of Biostatistics, Vanderbilt University Medical Center, Nashville TN 37203, USA.
2. Department of Biomedical Engineering, Vanderbilt University, Nashville TN 37235, USA.
3. Neuroscience Program, Vanderbilt University, Nashville TN 37235, USA.
4. Department of Ophthalmology and Visual Sciences, Vanderbilt University Medical Center, Nashville TN 37232, USA.

* These authors contributed equally to this work

SUPPLEMENTARY FIGURES

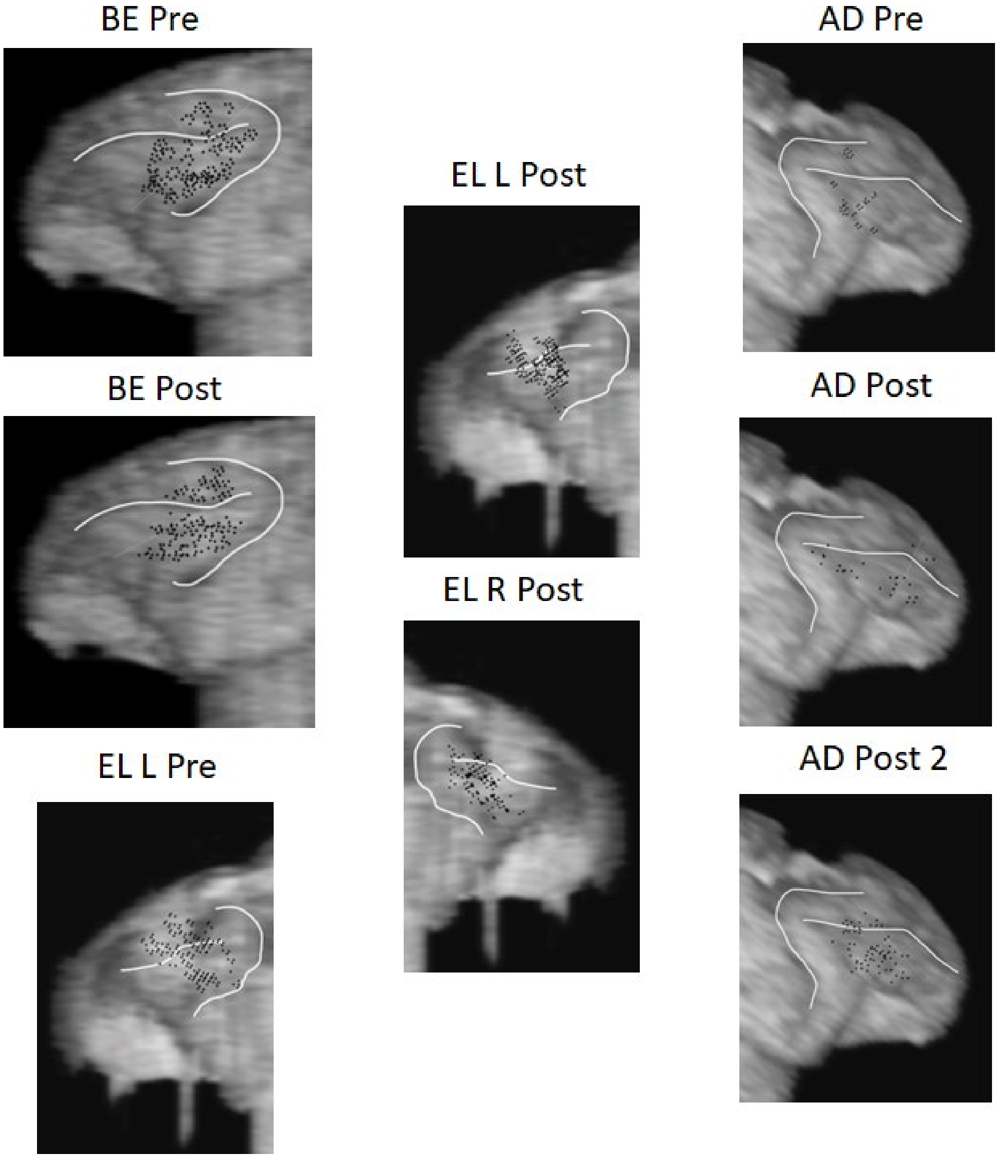

Figure S1. Anatomical localization of electrode penetrations in three monkeys (BE, EL, AD). Penetrations in pre-training and post-training recordings are shown, from the left (L) or right hemisphere (R).

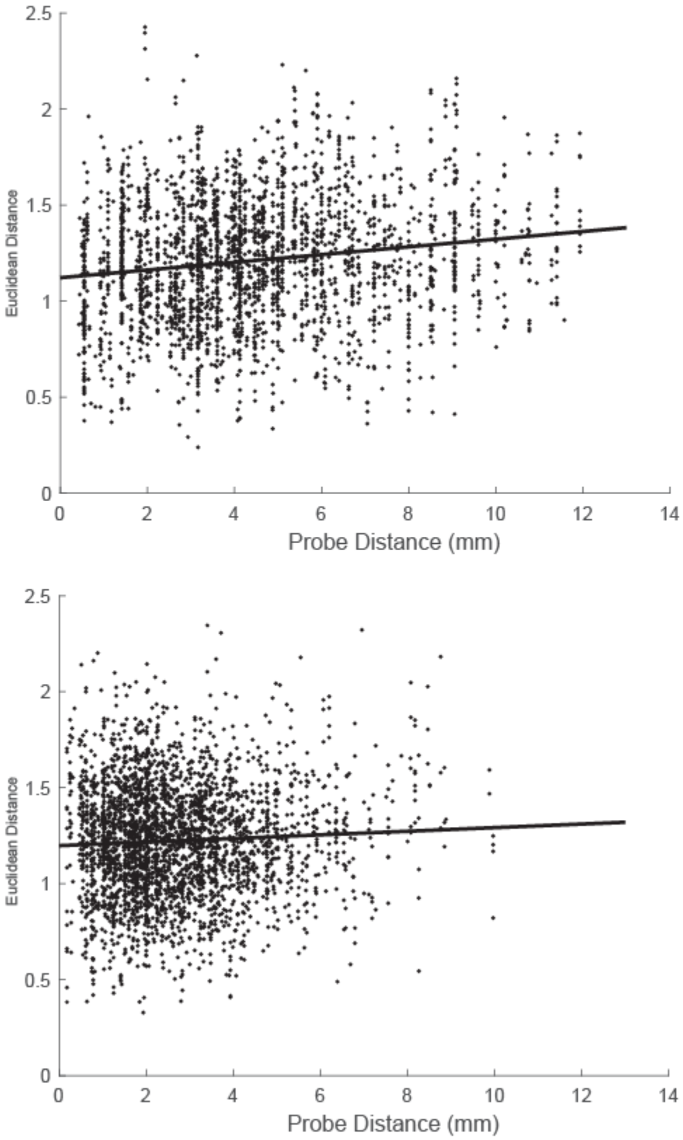

Figure S2. Euclidean distance between the preferred locations of the neurons in every possible pair as a function distance between the anatomical site where they were recorded (on different days). Top, data from the cue period, prior to training. Black line represents linear regression, p= 6.5e-16. Bottom, data from the cue period after training: p= 3.7e-03.
